# Therapeutic effects of stem cells in different body systems, a novel method that is yet to gain trust: A comprehensive review

**DOI:** 10.17305/bjbms.2021.5508

**Published:** 2021-12

**Authors:** Alireza Ebrahimi, Hanie Ahmadi, Zahra Pourfraidon Ghasrodashti, Nader Tanideh, Reza Shahriarirad, Amirhossein Erfani, Keivan Ranjbar, Soheil Ashkani-Esfahani

**Affiliations:** 1 Student Research Committee, Shiraz University of Medical Sciences, Shiraz, Iran; 2 Molecular Pathology and Cytogenetics Laboratory, Department of Pathology, Shiraz University of Medical Sciences, Shiraz, Iran; 3 Stem Cells Technology Research Center, Department of Pharmacology, Shiraz University of Medical Sciences, Shiraz, Iran; 4 Thoracic and Vascular Surgery Research Center, Shiraz University of Medical Sciences, Shiraz, Iran; 5 Department of Orthopaedic Surgery, Massachusetts General Hospital, Harvard Medical School, Boston, Massachusetts, USA

**Keywords:** Stem cell therapy, degenerative diseases, clinical and translational research, pluripotent cells

## Abstract

Stem cell therapy has been used to treat several types of diseases, and it is expected that its therapeutic uses shall increase as novel lines of evidence begin to appear. Furthermore, stem cells have the potential to make new tissues and organs. Thus, some scientists propose that organ transplantation will significantly rely on stem cell technology and organogenesis in the future. Stem cells and its robust potential to differentiate into specific types of cells and regenerate tissues and body organs, have been investigated by numerous clinician scientists and researchers for their therapeutic effects. Degenerative diseases in different organs have been the main target of stem cell therapy. Neurodegenerative diseases such as Alzheimer’s, musculoskeletal diseases such as osteoarthritis, congenital cardiovascular diseases, and blood cell diseases such as leukemia are among the health conditions that have benefited from stem cell therapy advancements. One of the most challenging parts of the process of incorporating stem cells into clinical practice is controlling their division and differentiation potentials. Sometimes, their potential for uncontrolled growth will make these cells tumorigenic. Another caveat in this process is the ability to control the differentiation process. While stem cells can easily differentiate into a wide variety of cells, a paracrine effect controlled activity, being in an appropriate medium will cause abnormal differentiation leading to treatment failure. In this review, we aim to provide an overview of the therapeutic effects of stem cells in diseases of various organ systems. In order to advance this new treatment to its full potential, researchers should focus on establishing methods to control the differentiation process, while policymakers should take an active role in providing adequate facilities and equipment for these projects. Large population clinical trials are a necessary tool that will help build trust in this method. Moreover, improving social awareness about the advantages and adverse effects of stem cell therapy is required to develop a rational demand in the society, and consequently, healthcare systems should consider established stem cell-based therapeutic methods in their treatment algorithms.

## INTRODUCTION

Stem cells are primal cells that are mutual to all multicellular organisms and can renew themselves through cell divisions, and differentiate into a wide range of specialized cell types [1].One of the main challenges for using therapeutic effects of stem cells is how to direct their differentiation process, decrease the immune rejection, and mitigate the risk of tumors derived from the transplanted stem cells [2]. To tackle these problems some novel technological interventions were proposed, for example, genetic modification of the stem cells, 3-dimensional bioprinting of the stem cells, and the addition of nanocomposites [3]. Numerous papers have been published discussing the ways to get stem cell therapy involved in the current treatment protocols of disease that might benefit from this method. The results are sometimes controversial and confusing though, deeming a comprehensive review of these potentials necessary.

In the present study, we have reviewed the literature reporting the stem cell-based treatments for a range of ­prevalent diseases in the society. We aim to provide a concise overview of the application of stem cell therapy on diseases of different body systems, including, cardiovascular, neurological, respiratory, gastrointestinal, and genitourinary systems.

### Classification of stem cells

**Figure 1:**
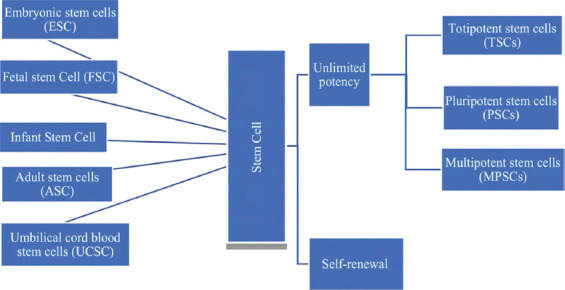
Stem cell potency and classifications.

The mammalian stem cells can be categorized into five broad categories (Figure 1).

#### Embryonic stem cells

Embryonic cell lines are derived from the epiblast tissue of the blastocyst structure, which is aligned in the inner wall of the blastocyst [4-6]. The blastocyst is a structure that forms in the early stages of embryo’s development, approximately during the 4^th^ to 5^th^ day of fertilization in humans. The blastocyst consists of 50-150 cells embryonic stem cells (ESCs) which are pluripotent and can transform to all derivatives of the three primary germ layers, including ectoderm, endoderm, and mesoderm. In other words, they can develop into each of the more than 200 cell types of the adult body, when given the sufficient and necessary stimulation for a specific cell type [4, 7-11]. ESC has the potency to divide *in vitro* after the administration of proper stimulant agents for differentiation, in which each daughter cell will also remain pluripotent. The pluripotency of ESC -*in vitro* and *in vivo*- has been repeatedly demonstrated in previous research [6, 11, 12]. Because of their unique capability of unlimited expansion, ESCs are perceived as a hypothetical source of cells in regenerative medicine and a proposed basis for tissue replacement in several diseases [4-8, 13].

#### Fetal stem cells

Fetal stem cells (FSCs) are cell lines that are derived from fetal tissues, which have the ability to divide, proliferate, and differentiate into specialized cells. Fetal stem cells can be isolated from fetal hematopoietic stem cells, fetal mesenchymal stem cells, and neural crest stem cells [14]. Studies demonstrated that FSCs have higher pluripotency potentials and lower immunogenicity effects compared to adult stem cells (ASCs) [14].

#### Infant stem cells

Infant (perinatal) stem cells can be derived from perinatal tissues, including, placenta membranes, amniotic fluid, and umbilical cords [15]. These tissues consist of numerous types of stem cells that possess the characteristics of both ESCs and ASCs [15]. Amniotic fluid stem cells, umbilical cord stem cells (UCSCs), and placenta-derived stem cells can be easily collected at the end of gestation. These stromal cells are considered to be the best candidates for stem cell therapy because they are the most abundant source of hematopoietic stem cells (HSCs) and mesenchymal stem cells (MSCs) [15].

#### Adult stem cells

ASCs or somatic stem cells are undifferentiated cells that can be found in post-natal adult tissues, which could be unipotent or multipotent [16, 17]. However, sometimes ASCs are known as progenitor cells, due to their less capability of cellular differentiation [8, 16, 17]. These cells are commonly classified into further categories as epidermal stem cells (EDSCs), neural stem cells (NSCs), MSCs, and HSCs [18].

#### Induced pluripotent stem cells

Induced pluripotent stem cells (iPSCs) are generated from somatic stem cells that have been reprogrammed into ESC-like state. iPSCs have the properties of ESCs, and can differentiate to the three primary germ layers. These cells have several advantages as they are derived from the patients’ own somatic cells leading to lower risks of rejection [18].

### Potentials of stem cells

Stem cells have the potential of self-renewal and differentiation as described below (Figure 1).

#### Self-renewal

Stem cells can undergo multiple cycles of cell division while preserving the undifferentiated state [19, 20].

#### Unlimited potency

Stem cells also have the capability to differentiate into any mature cell type. This makes stem cells either totipotent, pluripotent, or multipotent [19].

#### Totipotent stem cells

Totipotent stem cells (TSCs) are the outcome of the fusion of a sperm cell and an oocyte. The fertilized egg initiates a few divisions and forms cells that have totipotent properties. TSCs have the ability to differentiate into embryonic and extraembryonic types of cells [21, 22].

#### Pluripotent stem cells

Pluripotent stem cells (PSCs) are the descendants of totipotent cells and can differentiate into cells that construct the three germ layers of ectoderm, mesoderm, and endoderm which subsequently give rise to various system organs [21-23].

#### Multipotent stem cells

Multipotent stem cells (MPSCs) are among the major groups of stem cells which hold similar basic properties of all stem cells [24]. These cells can differentiate into various specific cells leading to particular properties, such as hematopoietic stem cells (HSCs), skeletal myoblasts, MSCs, and endothelial progenitor cells (EPCs) [25-27]. These cells can inhibit or activate a sequence of molecular and cellular pathways resulting in antiapoptotic and anti-inflammatory properties that can contribute to the treatment of various diseases.

#### Division and differentiation

Stem-cells have two types of cell division in order to remain self-renewed and potent. Firstly, a symmetric division resulting in two identical daughter cells both having the stem-cell potentials. The second type is called asymmetric division whose outcome is one stem-cell with the aforementioned properties and a progenitor cell with less self-renewal potential [9, 10, 28, 29].

### Therapeutic effects of the stem cells

**Figure 2:**
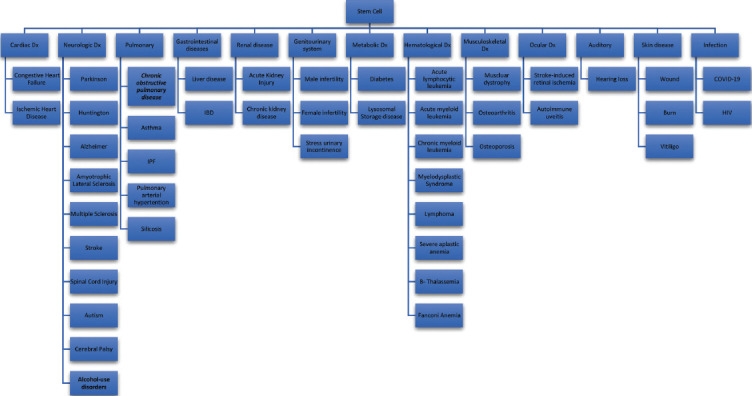
A review of stem cell therapy in various systems.

Stem cell therapy has been applied in various disorders in which here we review some of the diseases in this regard (Figure 2 and Table 1].

**Table 1:**
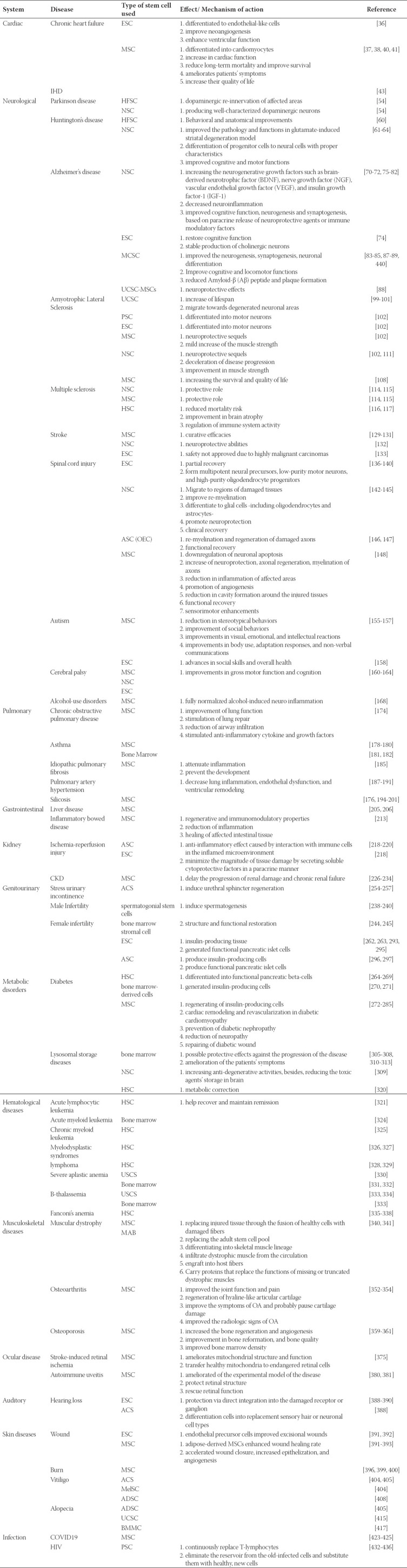
A review of stem cell therapy and effects.

#### Cardiac diseases

*Congestive Heart Failure (CHF) and Ischemic Heart Disease (IHD)*

Cardiovascular diseases (CVD) account for about 20% of the burden of noncommunicable diseases (NCD) [30]. These diseases are responsible for nearly one-third of deaths globally, and the prevalence of them are continuing to grow in both developing and developed countries [31]. Current treatments rarely could ameliorate the underlying causes of CVD, which are tissue scarring and cell loss [32].

Rising evidence demonstrated stem-cell therapy could improve heart failure by inducing neovascularization, decreasing cardiomyocyte apoptosis, and forming resident stem-cells [33-35]. Animal studies suggested that ESCs can be differentiated to endothelial-like cells, improve neoangiogenesis, and enhance ventricular function [36]. Besides, transplantation of bone-marrow-derived MSCs for myocardial infarcted animal models showed consistent results, as MSCs differentiated into cardiomyocytes [37, 38].

The first U.S. randomized controlled clinical trial, using a three dimensional guided catheter system to provide the heart with muscular stem-cells, was done in 2009; and after 1 year of follow-up, the results showed significant improvements in New York Heart Association (NYHA) and Minnesota Living with Heart Failure Questionnaire (MLHFQ) scores, besides the increase of ventricular viability [39]. Endomyocardial injection of cardiopoietic stem-cells (MSCs exposed to a cardiogenic cocktail) to the patients with preclinical heart failures showed the same results; as the patients had improvements in NYHA, 6-min walk distance, Quality of Life (QoL) and left ventricular ejection fraction [40]. Furthermore, 6 months follow-up of patients who had done subepicardial transplantation of autologous stem cells into ischemic myocardium demonstrated a major increase in cardiac function, measured by echocardiography, single-photon emission computed tomography, and angiography [41]. The STAR-heart study asserted that intra-coronary bone-marrow-derived MSCs transplantation in patients with chronic heart failure could ameliorate patients’ symptoms, increase their QoL, and improve survival [42]. Stem-cell therapy also can reduce the long-term mortality of patients with CHF and chronic IHD [43]. Moreover, it can reduce incidence of arrhythmias and non-fatal myocardial infarctions in the long-term in these patients [43]. The effects of stem-cell therapy on the rehospitalization and long-term left ventricular ejection fraction of patients with CHF and IHD are controversial, and more studies are needed to find concrete results [43]. To the best of our knowledge, the report of procedural adverse effects and side effects such as cardiac or systemic toxicity induced by stem cells are rare. Thus, the therapy could be perceived as a safe and feasible treatment for patients with CHF and IHD; however, the procedure must be applied cautiously and more clinical evidence is needed for a definite conclusion.

#### Neurological diseases

*Parkinson’s disease*

Parkinson’s disease (PD) is a neurodegenerative disease that is common worldwide and clinically demonstrates itself by symptoms of resting tremor, bradykinesia, rigidity, and postural instability [44, 45]. The pathology of the disease is characterized by loss of dopaminergic neurons in the pars compacta of substantia nigra (SNpc) and other areas of the brain, besides development of protein residues in neurons’ cytoplasm (Lewy bodies) [46, 47]. Present therapeutic options for PD patients consisted of deep brain stimulation and dopamine agonists.

Promising evidence has been found regarding neuroprotective and neuroregenerative benefits of stem-cell therapy –while applying with neurotrophic agents. Transplantation of embryonic mesencephalic into the brain of animal models showed improvement of dopamine transmission [48-50].

The proof-of-principle has been achieved by several studies in which evidence was in favor of the hypothesis that the dead dopaminergic neurons can be replenished by stem-cell transplantation in patients with PD [8, 51-53]. Most of the studies have been using human fetal stem-cells (hFSCs) of ventral mesencephalic neurons as the transplantation grafts; which can provide dopaminergic re-innervation of affected areas [54]. Foremost problems of using hFSCs are the low availability and absence of the cell material standardization which causes high inconsistency considering the degree of symptom relief, besides, the occurrence of adverse effects such as graft-induced dyskinesia in some patients [55-57].

Due to the aforementioned issues, the current researches emphasize the adjustment of hFSCs, and also producing well-characterized dopaminergic neurons by using ­brain-derived NSCs. Stem cell tissues –to be grafted in the striatum- must be analogous to substantia nigra neurons, regenerate the dopamine network in the brain, produce long-term and high symptomatic relief, and have no adverse effects [54]. It is still unclear whether the transplantation of stem-cell-derived neurons can replace dead compartments in PD patients; however; preceding studies on hFSCs provided us with the essential knowledge to establish clinically competitive stem cell therapy in this field.

*Huntington’s disease*

Huntington’s disease (HD) is an uncommon neurodegenerative disease characterized by undesirable motor and cognitive functions, besides psychiatric syndromes. The disease is considered a genetic disorder that is inherited in an AD (autosomal dominant) fashion and results in cell loss in the striatum and cerebral cortex [58]. Current treatments of HD are mostly based on symptom therapies –antipsychotic and antidepressant drugs- and hardly improve the underlying causes of the disease.

Several studies on animal models have suggested that stem-cell therapy could be used in the case of HD [59]. Behavioral and anatomical improvements have been seen after the transplantation of HFSCs into rodent striatum [60]. Moreover, intravenously injected NSCs have migrated into striatal lesions and improved the pathology and functions in the glutamate-induced striatal degeneration model [61]. The transplantation of adult NSCs in animal models also showed significant improvement of motor function impairments as progenitor cells could differentiate to neural cells with proper characteristics [62].

This accumulating evidence indicates that cell transplantation therapy could be well justified as a treatment of HD. Several studies experimented with the feasibility of cell therapy in this regard and showed promising results. Positron emission tomography (PET) scanning of 3 out of 5 patients who underwent hFSCs transplantation into the striatum demonstrated increased metabolic activity in the region; besides the patients showed improved cognitive and motor functions, through six years of follow-up [63, 64]. The same results were achieved in another study which asserted that 3 patients who underwent striatal transplantation of hFSCs showed increased scores of cognitive measures [65]. A previously done investigation approved the safety of intrastriatal neurotransplantation of hFSCs [66]. However, the main concerns about the use of hFSCs are the high tumorigenic properties of these cells, scarcity of this material, as well as ethical and moral issues.

*Alzheimer’s disease*

Alzheimer’s disease (AD) is the most common neurodegenerative disease which results in irreversible loss of neurons in the brain, mainly in the hippocampus and cortex [67]. The disease is diagnosed, clinically, by the symptoms of progressive demolition of memory, judgment, decision-making, and orientation [68]. Current AD treatments, such as the prescription of acetylcholinesterase inhibitors to enhance cholinergic function, provide an only partial and temporary alleviation of symptoms. The treatment of AD has been a daily challenge for physicians many years ago because of the preceding fact.

Multiple theoretical approaches have been proposed in order to design stem-cell therapy for AD such as the upregulation of the local NSCs, and stem-cell transplantation [69]. The upregulation of the neurons could be achieved through medicine and gene-therapy by increasing the neurodegenerative growth factors such as brain-derived neurotrophic factor (BDNF), nerve growth factor (NGF), vascular endothelial growth factor (VEGF), and insulin growth factor-1 (IGF-1]; although animal studies showed this approach lacks the potency to repair neurons [70-72].

Investigations on animal models of AD showed ESCs have the potential to restore cognitive function; however, no tangible clinical evidence has been succeeded yet [73]. A recent investigation reported stable production of cholinergic neurons from human ESC, which was able to functionally integrate into hippocampal neuronal circuitry, after transplantation [74]. The transplantation of NSCs has shown more promising outcomes as it decreased the neuroinflammation and improved cognitive function, plus, neurogenesis and synaptogenesis in aged primate models and AD mice replicas; these effects could be because of the paracrine release of neuroprotective agents or immune-modulatory factors, besides, the direct neural differentiation [75-82]. Moreover, investigations have demonstrated that the transplantation of MSCs into the brain of aged mice models have improved the neurogenesis, synaptogenesis, neuronal differentiation, plus, cognitive and locomotor functions, and reduced Amyloid-b (Ab) peptide and plaque formation; it has been also stated the intravenous administration of MSCs could also be effective by crossing the blood-brain barrier and migrating to areas of brain injury [83-89].

Recently, a phase 1 clinical trial showed that human umbilical cord blood-derived mesenchymal stem-cells (hUCB-MSCs) which injected by stereotactic technique into the brain of AD patients could have neuroprotective effects, as patients didn’t show a decline in their cognitive functions; besides, no adverse effects were observed during 24-months follow-up [90].

Given that the rate of neuroregeneration in the adult brain is minimal and that the pathology of AD consists of Ab formation from amyloid precursor protein (APP), Alzhimer’s patients have markedly reduced brain functions. Stem-cell transplantation therapy for AD may not be effective in that environment, where APP metabolism is altered, and it may, conversely, lead to excessive gliogenesis and tumorigenesis. Studies must consider the regulation of APP processing to develop effective stem-cell transplantation protocols for AD patients [91-95].

#### Amyotrophic Lateral Sclerosis

Amyotrophic Lateral Sclerosis (ALS) is a progressive neurodegenerative disorder that involves degenerations of motor neurons at all stages including the cerebral cortex, brainstem, and spinal cord [96]. The symptoms of the disease consist of the indications of loss of neurons at all levels of the motor system –both upper and lower motor neuron findings- which results in progressive muscular paralysis [96]. The progression of ALS is inevitable, and about 50% of patients die within 3 years of suffering from the disorder [97]. Currently, the condition is perceived as incurable and the treatments provide symptomatic relief in order to increase the QoL of patients. Stem-cell therapy has been under investigation to be used as a cure for the condition and has shown several promising pieces of evidence in this regard. The therapy could restore and preserve the function of both upper and lower motor neurons; besides, newly renovated neurons could be integrated into existing neural circuitries [21, 98].

Preclinical studies and treatment of animal models of ALS with human umbilical cord blood cells demonstrated the increase of lifespan in a dose-dependent way [99, 100]. Moreover, intravenous administration of these cells to the mice models showed stem cells tend to migrate towards degenerated neuronal areas [101]. iPSCs and human-derived ESCs are perceived as the two most powerful types of stem cells that could benefit ALS patients; MSCs and NSCs are also safer products as they do not have the adverse effects of prior cell types such as tumorigenesis [102]. Investigations have suggested that ESCs and iPSCs of ALS patients can be differentiated into motor neurons [103, 104]. MSCs and NSCs have been used in clinical trials and they have shown more neuroprotective sequels rather than neuroregenerative outcomes. Intrathecal and spinal cord injection of MSCs has been used in phase 1 clinical trials which showed a mild increase of the muscle strength with minimal adverse effects [105-107]. Furthermore, autologous-derived stem-cells injected into the frontal cortex of patients with ALS showed the potential of increasing the survival and QoL of these patients [108]. Phase I intraspinal injection of NSCs has also been done in patients with ALS, the disease progression was slowed and no severe adverse effect was observed during the examination [109, 110]. The result of another recent study was also consistent with prior findings as to the microinjections of NSCs into the lumbar cord tract showed an improvement in muscle strength in these patients [111].

*Multiple sclerosis*

Multiple sclerosis (MS) is an autoimmune, inflammatory, and chronic neurological condition that involves myelinated axons of the central nervous system (CNS) [112]. The definite underlying cause of the disease is yet to be cleared, however, a number of genetic and non-genetic factors have been mentioned to be the cause of the disease [112]. Currently, there is no curative therapy available for the disease and the treatments involve disease-modifying agents such as Beta Interferons, Glatiramer Acetate, Mitoxantrone, Natalizumab, and Fingolimod, besides symptomatic relieving drugs such as Dalfampridine along with a variety of off-label drugs [112]. The disease is a progressive condition and treatments can only partially manage the course of MS.

Previous studies have found that NSCs and MSCs administration may have a protective role against the progression of MS in both rodents and primates [113]. Several pilot studies have suggested that autologous MSC administration may be a feasible and safe method regarding the treatment of MS patients [114, 115]. Moreover, autologous HSC transplantation was also found to be beneficial in patients with progressive MS, as it significantly reduced mortality risk [116]. Multiple phases I/II studies showed that the transplantation of autologous HSC could have promising results such as improvement in brain atrophy that was evaluated by Magnetic Resonance Imaging (MRI), and regulation of immune system activity [117]. It has also been reported that a few MS patients that received allogeneic bone marrow transplantation because of a coincidental hematopoietic disorder had recovered from this immune-mediated disease [117].

*Stroke*

Stroke disorders are the major cause of morbidity in many areas of the world that could lead to irrecoverable brain tissue damages and functional disabilities. Current therapeutic approaches are mostly focused in order to modify risk factors for stroke such as hypertension, heart diseases, diabetes mellitus, heavy cigarette and alcohol consumption, and hypercholesterolemia [118]. Surgical interventions and intravenous administration of thrombolytic agents, as reperfusion therapies, are only available in the acute stage of the stroke and cannot be done on many occasions. Recently, physicians have suggested that brain function may improve after stroke by the restoration of damaged neuronal cells through stem-cell transplantation.

A variety of precursor cell types such as human bone marrow cells, neuroepithelial stem cell line, NTera-2 cell line have been investigated to renovate brain function after stroke in animal models of stroke which showed improvement of sensorimotor function, spatial memory, neurological severity score, and asymmetrical motor behavior [119-128]. Although, the underlying causes of these improvements are yet to be cleared and there are few indications of neuronal replacement. Autologous somatic stem cells such as MSCs appear to be a reasonable source in this field since the chance of graft rejection is minimal. However, the plasticity of somatic stem cells is not obvious and it is not clear that they can safely evolve into neuronal cells [129]. In this regard, intravenous administration MSCs was done in stroke patients and its safety plus curative efficacies were observed during long-term follow-up [130, 131]. Moreover, studies have suggested that endogenous pools of stem cells, for example, NSCs could also be considered as a source for stem-cell therapy because of their neuroprotective abilities [132]. It is worth mentioning that pieces of evidence from a study on animal models of stroke did not approve the safety of ESCs transplantation in this field, as ESCs produced highly malignant carcinomas at the site of implantation [133].

The curative effects of stem-cell therapy on animal models of stroke made the basis for the treatment to be addressed for use in human subjects. Initial clinical trials showed that stem-cell therapy could be safe, feasible, and therapeutic in patients with stroke; however, more comprehensive is needed to approve the aforementioned issue.

*Spinal cord injury*

Spinal cord injury (SCI) is a destructive condition that involves both upper and lower motor neurons and could result in paraplegia and quadriplegia. The incidence of traumatic SCI, primarily caused by motor vehicle accidents, is estimated to be between 28 and 55 per million people each year, in the US [134]. The management of the condition consists of both surgical –for example, surgical decompression and spine stabilization- and medical –for instance, steroid therapy- interventions; besides rehabilitation and long-term follow-up of the patients [134]. Recently, several other newly designed approaches have been suggested in this regard such as molecular and cellular therapies. [135]. The cellular therapy of the condition focuses on stem/progenitor cells and transplantation of Schwann cells, peripheral nerve cells, and cells from the olfactory nervous system [135].

The emerging perspective of stem cell therapy as a viable therapeutic tool for the treatment of SCI is based on the preclinical studies which have been performed on animal models and have shown that transplanted progenitor cells can migrate into the injured tissues to improve axonal regeneration and motor function. ESCs have been used for the treatment of paralyzed rats, the paralyzed rodents showed partial recovery from the condition as the transplanted ESC–derived axons restored SCI [136]. Moreover, ESCs were also able to form multipotent neural precursors, low-purity motor neurons, and high-purity oligodendrocyte progenitors [137-141]. NSCs have been proposed as a possible treatment of SCI; because they are already devoted to a neural fate and have a lower potentiality to be neoplastic. Several preclinical investigations demonstrated that transplanted NSCs could reach regions of damaged tissues, improve re-myelination, differentiate to glial cells -including oligodendrocytes and astrocytes-, promote neuroprotection, and cause clinical recovery in animal models [142-145]. Olfactory ensheathing cells (OECs) were also used in rodent models of SCI and caused significant remyelination and regeneration of damaged axons, besides functional recovery [146, 147]. Large production of the prior cells is moderately difficult, and it is not clear whether they can be increased to an adequate amount to be administered in human clinical trials. MSCs have been widely used in the case of SCI and the results were drastically positive regarding the treatment of the condition. Preclinical studies suggested downregulation of neuronal apoptosis, an increase of neuroprotection, axonal regeneration, myelination of axons, reduction in inflammation of affected areas, promotion of angiogenesis, reduction in cavity formation around the injured tissues, besides functional recovery, and sensorimotor enhancements, after the transplantation MSCs in animal models with SCI [148].

NSC transplantation is under investigation at phase I/II clinical trial and is proved to be safe and feasible for perilesional intramedullary injections [145, 149]. The grafting of MSCs in humans also resulted in partial functional and behavioral recovery [148]. However, a recent phase 3 clinical trial showed controversial results as only 2 among the 16 patients showed progress in neurological status after MSCs application (150].

Briefly, animal models of SCI who received stem-cell therapy have demonstrated several promising results, and preclinical investigations have validated scientific principles and approaches. Additionally, multiple signs of progress have been made by a number of studies with practical concerns in this regard.

*Autism*

Autism and autism spectrum disorders are referred to as neurodevelopmental disorders that are caused by both genetic and environmental factors and are characterized by disruption of social interactions and stereotypic behaviors [151-153]. Current therapeutic interventions for autistic patients include behavioral and psychosocial treatments, besides, psychopharmacological treatments that could not completely resolve the patients’ symptoms [154].

Recently, researchers have conducted several investigations regarding the use of stem cell therapy for autism. Intracerebroventricular transplantation of MSC for animal models of the disease showed promising results, as a reduction in stereotypical behaviors and an improvement of social behaviors were observed during the investigation in animal models who received the transplantation [155].

Consistently with prior results, a phase I/II clinical trial showed that the patients who received the transplantation of human cord blood mononuclear cells and umbilical cord-derived MSCs had improvements in visual, emotional, and intellectual reactions, along with body use, adaptation responses, and non-verbal communications [156]. Autologous bone marrow mononuclear cell transplantation was also found to be useful regarding the treatment of autistic patients [157]. Another pilot study has also demonstrated the beneficial effects of ESC administration for autistic patients, as significant advances in social skills and overall health were observed after the treatment [158]. Moreover, the treatment safety was evaluated and no serious adverse effect was observed during stem cell transplantation in the mentioned patients [156, 158].

*Cerebral palsy*

Cerebral palsy (CP) is a group of chronic neurological diseases that result in cognitive and motor impairments in children. This may be caused by perinatal infection, asphyxia, and preterm birth, or even postnatal brain injuries [159]. The overall incidence of CP is increasing which could be due to the increased rate of premature infants’ survival [159]. Currently, available treatments for CP include rehabilitation strategies and pharmacological interventions to reduce the symptoms.

Several lines of stem cells such as MSCs, NSCs, and ESCs have been administered to CP patients in previous investigations [160-164]. Stem cell intervention could be a promising alternative treatment for pediatric patients with CP according to the conducted clinical trials. Previous studies demonstrated that stem cell therapy could result in significant improvements in gross motor function and cognition as well [165]. The adverse effects of the stem cell administration in these patients were reported to be at a minimum [165]. However, it has been mentioned the beneficial effects of stem cell interventions in CP patients may be for short-term periods, and in order to achieve comprehensive results, more investigations must be conducted in this regard.

*Alcohol-use disorders*

Alcohol-use disorders have been reported to be responsible for 1 out of 10 deaths among working-age adults in the US and are also among the leading causes of morbidity and premature mortality worldwide [166]. Also, chronic alcohol consumption is known to be the most common reason for peripheral and central nervous system toxicity [167]. Animal experiments have demonstrated that a single dose of MSC-spheroids can have a significant beneficial effect on the animal model of chronic ethanol intake and relapse-drinking by inhibiting chronic ethanol intake, and subsequently, fully normalized alcohol-induced neuroinflammation [168-171].

#### Pulmonary diseases

*Chronic obstructive pulmonary disease (COPD)*

COPD has been reported to be the third-leading cause of mortality in the US [172, 173]. Regardless of recent developments in the management of symptoms with novel pharmaceutical medications and molecules, no effective treatment has yet been obtained in order to attenuate or reverse the disease progression or emphysematous changes.

Meta-analytic studies on animal models of COPD have shown that intra-tracheal instillation or intravenous injection of MSC have satisfactory results, through the significant improvement of lung function and stimulation of lung repair. Furthermore, a considerable reduction of airway infiltration by macrophages, neutrophils, and products of pro-inflammatory cytokines such as IL-6 and IL-1b, but accompanied with substantial stimulated anti-inflammatory cytokine IL-10 and growth factors such as EGF, HGF, TGF-b, and VEGF was detected through MSC administration, suggesting this method as an effective approach in the treatment of animal models of COPD/emphysema, and therefore hold the foundation of further research and application of administering MSC in COPD patients [174].

*Asthma*

Asthma, affecting around 300 million individuals worldwide, is known to be primarily a chronic inflammatory disease presenting with lifelong conditions with different levels of severity [175, 176]. While the present treatments are effective for the management of asthma, patient’s morbidity, and mortality are still factors requiring attention, which continue attempts in modifying and adjusting therapeutic options are being practiced [177]. There are increasing studies that show the beneficial effects of MSC-derived secretome in the treatment of experimental asthma [178-180]. Moreover, the use of bone marrow-derived mononuclear cells (BMMC) in animal models of severe asthma showed promising results [181, 182].

*Idiopathic pulmonary fibrosis*

Idiopathic pulmonary fibrosis (IPF) with a prevalence from 4.6 to 16.3/100,000 cases worldwide is characterized by chronic, progressive fibrosing interstitial pneumonia without a known etiology [176, 183]. Despite several therapeutic methods, no significant decrease in mortality rates have been observed [184].

There is evidence that MSCs can prevent the progression of animal model IPF and decrease inflammation, if administered early during the disease, however such evidence during the latent and established fibrosis period of the disease is limited and requires further studies in order to apply in clinical studies [176, 185].

*Pulmonary arterial hypertension*

Pulmonary arterial hypertension (PAH) is considered a rare and progressive disease which is characterized by the elevation of blood pressure of the lung arteries. While manageable, no effective therapeutic option has yet been approved in order to reduce mortality [186].

Animal studies have demonstrated that administration of MSCs, either intravenous or intratracheal, can decrease factors associated with PAH, including lung inflammation, endothelial dysfunction, and ventricular remodeling [187-191]. Therefore, these pieces of evidence can be the theoretical basis for designing future clinical trial studies.

*Silicosis*

Silicosis is among the known occupational diseases which cause extensive lung fibrosis and subsequently respiratory failure [192]. Currently, there are no promising clinical treatments to decelerate or halt the progression of the disease [193]. However, MSCs have shown to apply therapeutic properties in lung diseases and represent an alternative treatment for silicosis, particularly adipose-tissue-derived mesenchymal stromal cells [176, 194-201].

#### Gastrointestinal Diseases

*Liver disease*

Liver injury can be subsequent to causes such as ischemia, cytotoxic, toxic–metabolic, or other insults causing tissue inflammation and therefore result in innate and adaptive immune responses which in turn can also directly cause additional injury [202]. The currently accepted treatment for end-stage liver disease is liver transplantation, a procedure which has its own challenges. [203, 204]. However, considerable results regarding cell-based therapy using MSCs have been demonstrated *in vitro*, *in vivo*, and even in clinical studies for the treatment of liver diseases [205, 206]. Nevertheless, there is still little evidence regarding the efficiency compared to conventional treatments and therefore further studies and clinical trials are justified [207-209].

*Inflammatory bowel disease*

Inflammatory bowel disease (IBD), which consists of Crohn’s disease (CD) and ulcerative colitis (UC) is assumed to be due to inappropriate response of the host’s immune system to intestinal microbes [210]. Standard treatments for the disease consist of immunosuppressive drugs, such as biologicals, with low or even no response rates [211, 212]. Hence, alternative therapeutic remains an unmet need. Based on reported literature, stem cell transplantation in IBD patients has demonstrated satisfactory therapeutic potential along with a low risk of adverse effects. In this regard, MSCs have immunomodulatory and regenerative properties, which subsequently leads to a decrease in inflammation along with the healing of affected intestinal tissue [213].

#### Kidney diseases

*Acute kidney injury*

Acute kidney injury (AKI) is characterized by stimulation of the immune system and elevated oxidative stress which can be caused by ischemia-reperfusion injury (IRI) [214-217]. Stem cell therapy has been proven effective in experimental models of AKI based on decreasing the degree of tissue injury via soluble cytoprotective factors in a paracrine manner, and also based on the anti-inflammatory effect subsequent to the interaction with immune cells in the inflamed microenvironment [218-222]. However, more translational studies from animal models are required to ensure their safety for application in clinical studies [223].

*Chronic kidney disease and end stage renal disease*

In renal injury, the progression to end-stage renal disease (ESRD) is presently only partially understood due to its complexity [224]. There are controversial reports regarding studies of stem cells in this area. While some studies have reported that MSCs would delay the progression of renal damage and chronic kidney disease (CKD) [225-233], other studies have reported MSCs to be ineffective [229, 230, 234].

#### Genitourinary diseases

*Male Infertility*

Infertility is the inability to achieve pregnancy despite one-year attempts, in which the rate of related factors for male infertility alone has been reported from 6.4% to 42.4% in studies [235, 236]. Various treatment methods have been applied such as optimization of sperm production, relief of obstruction, and surgical extraction of sperm [237]. Treatment with stem cells by utilizing spermatogonial stem cells (SSCs) has been evolving in order to induce spermatogenesis from the individuals’ undifferentiated or dysfunctional SSCs [238-240]. However, caution should be warranted in this therapeutic option regarding carcinogenesis and also genetic/epigenetic alterations in offspring which should be fully investigated in humans [241].

*Female infertility*

The incidence of female infertility ranges from 33 to 41% worldwide [242]. The US reported that in 2002, 7.4% [2.1 million) of married women were infertile [243]. These statistics highlight the notable numbers of females enduring infertility.

Among the many causes of female infertility, reproductive system-related diseases are considered the main causes. The efficacy of bone marrow stromal cells has been demonstrated in several animal model studies regarding the treatment of chemotherapy-induced ovarian failure, indicating structure and functional restoration [244, 245]. Studies have shown restored menstruation in endometrial injury by the implantation of autologous bone marrow stromal cells [246]. Bone marrow stromal cells have also been successful in treating thin endometrium in animal model studies via locating the differentiation site in the tissue and exerting immunomodulatory properties [247]. Furthermore, functional endometrium restoration and improved reproductive outcomes have been detected in patients with Asherman syndrome using bone marrow stromal cells [247-249].

*Stress urinary incontinence*

Stress urinary incontinence (SUI) is a common urinary system disease affecting more than 200 million people worldwide and notably females, resulting in a serious decrease in QoL with no ideal therapeutic option until now [250-253]. Stem cell therapy has demonstrated promising results, by inducing urethral sphincter regeneration utilizing bone marrow-derived MSCs, muscle-derived stem cells, urine derived stem cells, and adipose-derived stem cells (ADSC) [254-257]. However, administration route and optimal timing of the stem cell requires further evaluation [258-260].

#### Metabolic disorders

*Diabetes*

Diabetes is a group of metabolic disorders that are characterized by hyperglycemia which is developed for a defect in insulin production, insulin function, or both. Diabetes mellitus (DM) is considered as one of the most common non-communicable diseases worldwide, with a global growing prevalence [261]. Persistent and uncontrolled hyperglycemia of diabetes may have severe consequences including retinopathy, neuropathy, and nephropathy if remains untreated. Currently approved treatments of DM include lifestyle and dietary modifications, prescription of anti-hyperglycemic agents, insulin, and some alternative medicines. However, these therapeutics could not properly improve the underlying cause of the disease which is the degeneration of pancreatic beta-cells, and in most cases, the disease could not be cured completely [261].

Several investigations have been done regarding the therapeutic use of both ESCs and ASCs in DM and its complications. ESC-derived insulin-producing tissue alleviated diabetes in mice [262, 263]. HSCs were also differentiated into functional pancreatic beta-cells in rodent models [264-269]. *In vitro* studies have also revealed the possibility of therapeutic administration of bone marrow-derived cells for DM patients, as these cells have generated insulin-producing cells (IPCs) under appropriate circumstances [270, 271]. Similarly, the advantages of MSCs were noticed in multiple studies as they could be beneficial in regenerating of IPCs, cardiac remodeling, and revascularization in diabetic cardiomyopathy, prevention of diabetic nephropathy, reduction of neuropathy, and repairing of the diabetic wound [272-285]. Moreover, other stem-cells such as those that originated from pancreatic duct, pancreatic islet, and exocrine tissue of the pancreas, besides, hepatic progenitor tissue were also investigated in this regard and promising results have been found [286-292]. Several *in vivo* and *in vitro* studies have been conducted to produce IPCs and functional pancreatic islet cells from human ESCs and ASCs [293-297]. The result of these experiments was most promising as fully functional beta and islet cells were generated in several laboratories.

In humans, pancreatic islet transplantation was suggested as a therapeutic approach through DM over two decades ago [298]. After the intraportal infusion of pancreatic islets in DM patients, approximately 50% reduction in insulin dependence was observed [299, 300]. Consequently, beta cell replacement therapy was also suggested and experimented as an alternative treatment for diabetic patients [301]. Previous studies have evaluated the efficacy of stem-cell therapy for patients with DM by using various methods such as umbilical cord blood transplantation, umbilical cord MSC therapy, HSCs infusion, and bone marrow MSC transplantation. These studies have suggested that stem-cell therapy could be a promising alternative treatment regarding the DM and its complications [302, 303].

*Lysosomal storage diseases*

Lysosomal storage diseases (LSDs) is an inherited defect in normal lysosomal functions which are identified by several different pathophysiologies [304]. LSD may have a variety of symptoms according to its pathological severity, including, mental alterations, cognitive retardations, rapid onset disabilities, and death. Several treatment methods have been suggested for these conditions which mostly focus on the replacement of the malfunctioned enzyme that is the underlying cause of the disease [305].

Studies on animal models of LSDs have shown the possible protective effects of bone marrow transplantation (BMT) against the progression of the disease [306-308]. Moreover, animal studies also demonstrated that intracranial injection of NSCs could have beneficial effects in increasing anti-degenerative activities, along with reducing the toxic agents’ storage in rats’ brains [309].

Allogeneic BMT was firstly done over 3 decades ago for patients who suffer from LSDs which showed promising results regarding the amelioration of the patients’ symptoms [305]. Later, this finding was evaluated by more expanded investigations which demonstrated that BMT could alleviate the patients’ symptoms in most cases [310-313]. Regarding the LSDs, BMT was found to be a feasible treatment in Hurler Syndrome, Maroteaux-Lamy Syndrome, Gaucher disease, Metachromatic leukodystrophy [314-319]. Furthermore, HSC transplant has been found to be more effective than enzyme replacement therapy in LSD patients, based on the metabolic correction [320].

#### Hematological diseases

*Acute lymphocytic leukemia*

Acute lymphocytic leukemia (ALL) is an aggressive malignancy that the lymphoid line of blood cells tend to be immaturely produced in large numbers. The main therapeutic approaches through this condition are chemotherapy and HSC transplantation that could help the patients to recover and maintain remission [321]. Currently, the main concern in this regard is finding the optimum strategy of the HSC administration [322, 323].

*Acute myeloid leukemia*

The main characteristics of acute myeloid leukemia (AML) are a rise in the number of immature myeloid cells which could result in several hematopoietic insufficiencies and leukocytosis. Stem cell transplantation has been administered for this condition since over 3 decades ago, however, there is still uncertainty regarding the optimal treatment of AML [324].

*Chronic myeloid leukemia*

Chronic myeloid leukemia (CML) is another malignancy of bone marrow cell which is characterized by an increased count of white blood cells in peripheral blood. It has been noticed in previous studies that CML is the most common indication of allogeneic HSC transplantation globally [325].

*Myelodysplastic syndrome*

Myelodysplastic syndromes are a group of bone marrow disorders that the blood cells are not properly matured. The main treatment of this condition is chemotherapy and HSC transplantation [326, 327].

*Lymphoma*

Another group of blood cancer is lymphoma which originated from the lymphatic system and could affect the whole body. The treatment of lymphoma is commonly chosen according to the type and severity of the disease. Radiation therapy, chemotherapy, immunotherapy, and HSC transplantation are considered as the main therapeutic options in this regard [328, 329].

*Severe aplastic anemia*

Severe aplastic anemia (SAA) is a condition characterized by the failure of bone marrow to make enough mature blood cells. Stem cell transplantation is a promising therapeutic approach through SAA; as previous investigations mentioned that unrelated cord blood transplantation and allogeneic bone marrow transplantation can be a salvage procedure for SAA patients [330-332].

*B-thalassemia major*

B-thalassemia is an early-onset and inherited blood disease which is characterized by severe anemia requiring regular blood transfusion. The main treatments of this condition consist of transfusion therapy, chelation therapy, surgical interventions such as splenectomy and cholecystectomy, and stem cell transplantation [333]. Bone marrow transplantation, umbilical cord blood transplantation, and combined transplantation of these two cell lines have been administered for patients with B-thalassemia major which showed satisfactory results [333, 334].

*Fanconi’s anemia*

Fanconi’s anemia (FA) is a rare genetic blood disorder that is caused by bone marrow failure to produce blood cells. HSC transplantation has been suggested as an effective and exclusive therapeutic intervention for these patients [335-337]. However, HSC transplantation may have some adverse effects which should be managed and reduced, including developing cancerous tissues and graft versus host disease [338].

#### Musculoskeletal diseases

*Muscular dystrophy*

Muscular dystrophy (MD) is among the muscle wasting diseases, and is a condition affecting a million individuals worldwide which are the result of mutations in structural proteins and are presented with loss of functional muscle subsequent to muscle fiber injury, deposition of fibrotic tissue, and inflammation [339]. Present therapeutic options are mostly supportive and address the inflammatory response.

The use of stem cell therapy has demonstrated evidence of replacing injured tissue through the fusion of healthy cells with damaged fibers along with replacing the ASC pool for long-term muscle maintenance [340]. Based on the potential of MSCs and mesoangioblasts (MABs) in differentiating into skeletal muscle lineage, Studies on animal models of MD have addressed these cells for the regeneration of injured skeletal muscle. These cells can also infiltrate dystrophic muscle from the circulation, engraft into host fibers, and bring with them proteins that replace the functions of those missing or truncated. Based on the systemic delivery property of these cells, an increase in the feasibility of stem cell therapy has been observed regarding a large number of affected skeletal muscles among muscular dystrophy patients [341].

*Osteoarthritis*

Osteoarthritis (OA) is the most prevalent joint disease globally, and it has been estimated that about 10% of men and 18% of women with the age of 60 or older suffer from the condition [342]. The pathology of the disease is mostly based on cartilage degradation which could be due to the activation of matrix proteases [343]. In severe cases, the pain and loss of function of the joint may inhibit the patients from doing their own daily activities, and decrease the quality of life (QoL) of the patients. Typically, the treatments of the condition consist of pain management, along with physical and occupational therapies, and joint replacement in end-stage patients [344-348]. Intra-articular injections of several agents such as hyaluronic acid (HA), platelet-rich plasma, hypertonic dextrose prolotherapy, and anabolic cartilaginous mediators have also been part of clinical trials attempting to cure OA [349-351]. The potentials of stem-cells, for instance, the capacity for regeneration of damaged tissues and reversing the chronic degenerative conditions, makes stem-cell therapy as an approach to setback the underlying causes of OA.

A 2-year follow-up, proof-of-concept trial showed that intra-articular injection of MSCs into OA knees improved the joint function and pain without any adverse events by regeneration of hyaline-like articular cartilage [352, 353]. Moreover, a systematic review published in 2018 asserted that stem-cell therapy could improve the symptoms of OA and probably halt cartilage damage [354]. The administration of MSCs has also improved the radiologic signs of OA [354]. The adverse effects after the MSC therapy were rare, and the most important ones were synovial effusion which needed overnight observation, and unstable angina in a case who had multiple risk factors after three months, plus pain and swelling [321, 355-357].

Currently, there are over 500 clinical trials registered on ClinicalTrials.gov, exploring the safety and efficacy of stem cells in order to find a comprehensive protocol to cure OA. Long-term follow-up is needed to evaluate the exact effects of stem-cells therapy in the condition.

*Osteoporosis*

Osteoporosis is considered the most common skeletal disease which is characterized by decreased bone mineral density and bone structural deformations, which is probably caused by an acceleration in bone resorption by osteoclasts [358]. Current therapeutic interventions for patients with osteoporosis include bone-resorbing drugs which may have several adverse effects.

The application of MSCs has increased bone regeneration and angiogenesis in osteoporotic mice [359]. Osteoporotic mouse models that were treated with a single dose of systemically injected MSCs showed an improvement in bone reformation, and bone quality [360]. A recent meta-analysis on preclinical studies has approved the rationale to conduct further clinical studies regarding the use of stem cell interventions as a treatment of osteoporosis, since the available investigations in animal models mostly showed an improved bone marrow density after the application of stem cells [361]. Furthermore, previous studies have proposed that administration of MSCs could be a promising treatment for osteoporotic patients [362-365]. However, it was also noted that the MSCs have a tendency to differentiate into adipose tissue in these patients; and this problem must be solved in future investigations [362, 366].

#### Ocular diseases

*Stroke-induced retinal ischemia*

Among the complications of cerebral ischemia one can name its effect on the retinal, causing cell loss and optic nerve damage [367-369]. Available approved treatments consist of either endovascular thrombectomy or tissue plasminogen activator (tPA) which include limitations such as the increased risk of further damage and short therapeutic window [370-372].

There is rising evidence that MSCs can significantly affect the pathogenesis of the disease [373, 374]. Both in vitro and in vivo treatment with MSCs have shown to transfer healthy mitochondria into injured retinal cells, resulting in the amelioration of mitochondrial function and structure after ischemia [375]. Combinational treatment along with enhancing the timing of stem cell transplantation and delivery route may have additional beneficial effects on recovering cell damage and visual loss and increase functional recovery, which requires additional investigation [376].

*Autoimmune Uveitis*

Autoimmune uveitis (AU) is among the leading reasons for visual disability. Current treatment options consist of long-term use of corticosteroids and immunosuppressive medicines, which in turn leads to serious side effects and possible progress of cataract and glaucoma; therefore, new and alternative therapeutic options are urgently needed [377, 378]. Recently, MSC-derived exosomes have shown promising therapeutic results in animal models in which periocular injection of MSC-derived exosomes considerably ameliorated experimental autoimmune uveitis, salvaged retinal function and protected retinal based on a clinical and histological basis [379-381]. However, there are still many challenges to be addressed in order to proceed with MSC-derived exosomes to be utilized as an effective therapeutic agent in the treatment of AU.

#### Auditory diseases

*Hearing loss*

Hearing loss by affecting more than 360 million people worldwide is among the diseases with a significant impact on the individuals’ physical and mental health [382-386]. The current effective treatment is cochlear implants which offer partial recovery at the expense of loss of residual hearing [387]. Stem cells have demonstrated potential in providing protection through direct integration into the damaged ganglion or receptor followed by differentiation into replacement neuronal cell types or sensory hair [388-390].

#### Skin diseases

*Wound*

The cutaneous wound is still considered as a global health care concern and its treatments include topical antibiotics, debridement, and application of grafts. There is growing evidence regarding the promising effect of stem cell therapy in wound healing. Both ESCs and MSCs were found to have wound healing properties. As human ESC-derived endothelial precursor cells improved excisional wounds in rats, and adipose-derived MSCs enhanced wound healing rate in animal models of DM [391, 392]. Furthermore, the administration of MSCs for wounds was found to result in accelerated wound closure, increased epithelization, and angiogenesis [393].

*Burns*

Burns are considered to be one of the most prevalent public health problems, causing high rates of morbidity and mortality particularly in low- and middle-income countries [394]. Finding a concrete approach in order to heal chemical, electrical, and thermal burns has always been a challenge for physicians. Commonly, surgical interventions (debridement and application of skin grafts) and pharmacological interventions (for example, silver sulfadiazine) are being suggested for patients with burn injuries. However, these therapeutic approaches often fail to prevent uncontrolled consequences of the trauma, such as scar formation and contracture, and neuropathies. Subsequently, researchers started investigating other treatment options, including administration of small interfering RNA, DNA, growth factors, and stem cells for burn injuries [395].

Stem cell therapy could be a promising intervention regarding burn injuries as previous investigations have shown the beneficial effects of EDSCs, MSCs, and ADSCs in wound healing [396]. Critical assessments of preclinical studies have demonstrated that stem cells significantly promote burn healing rate and wound healing time, with hair follicle stem cells (HFSC) having the highest efficacy [395, 397]. The application of stem cells has also resulted in increased angiogenesis and reduced inflammatory markers in burn wounded animals [395]. Besides, intravenous administration of ADSCs was found to be safe and effective in improving skin graft elasticity, in severely burned porcine models [398]**.** Having said that, several studies suggested that the administration of MSCs could be applied as an alternative treatment for chronic non-healing wounds [399, 400]. The results of clinical trials were also consistent with above-mentioned evidence, such as the vascularization and granulation of the wound, and the patients’ pain levels were considerably improved after administration of MSCs [397]**.** Moreover, prior clinical trials noticed that stem cell therapy could significantly decrease the scar formation and skin contracture [401]. Overall, it seems that stem cell therapy could improve burn wound healing, however, more clinical studies are needed to determine the safety and efficacy of this approach toward the problem.

*Vitiligo*

Vitiligo is a depigmenting condition of the skin caused by the loss of melanocytes that globally affects about 2% of the general population [402]. Several hypotheses were asserted to be the pathology of the condition, however, there is no definite explanatory hypothesis in this regard. The most widely accepted hypothesis is that both hereditary and non-hereditary factors, affecting the melanocyte function, can lead to immune-mediated destruction of melanocytes [403]. Currently, therapeutic approaches consist of the use of local and intralesional prescription of corticosteroids, plus psoralen ultraviolet light therapy [402].

The skin is composed of several populations of stem-cells such as EDSCs, HFSCs, and dermal mesenchymal stem cells [404, 405]. Moreover, the outer root sheath of the hair follicles is an undeniable source of melanocytes stem cells (MelSCs) [404]. Subsequently, the idea of reactivating these cells has been the fundamentals of some available treatments, including phototherapy. The MelSCs have been shown to be effective in the recovery of vitiligo, as they can initiate repigmentation, and some therapeutic approaches such as tacrolimus prescription, phototherapy, and dermabrasion help the condition to be cured by reactivation of MelSCs [404]. Recently, autologous transplantation has been used for treatment of vitiligo, whereas, it is relied on MelSCs which are difficult to be cultured and amplified, *in vitro* [403]. So that it has been suggested that future preclinical and clinical investigations might be needed to consider and investigate other progenitor cells, including, MSCs, ESCs, and iPS [403, 406]. In this case, prior studies have mentioned that MSCs could promote the regimentation process as they regulate the underlying immune response leading to the disease [407]. ADSC were also investigated as supporting graft in animal models that received MelSC transplantation; the co-transplantation of ADSCs and MelSC had more beneficial effects in comparison with monoculture MelSC [408].

*Alopecia*

Alopecia is one of the most common concerns of many individuals who come for medical consultations, and is caused by reduced hair follicle regenerative functions. The prevalence of the condition is estimated to be high in many regions of the world; for example, it has been calculated that 2% of individuals might suffer from alopecia areata (AA) and androgenetic alopecia (AGA) could be diagnosed in 31% (40-55 years old) to 53% (65-69 years old) men [409, 410]. Considering the psychological impacts of hair loss, clinicians have examined various therapeutic approaches aimed at halting or curing this condition, including, administration of corticosteroids or immunosuppressive agents, topical minoxidil, topical immunotherapy, and low-level laser light therapy [411]. While these therapeutics have been found to be effective to some extent, there’s still a demand for non-surgical curative options, as the patients do not always see that the hair had been significantly restored [412, 413]. Stem cell therapy might be the option that many seek for their hair loss, and be the appropriate treatment for resistant AA and AGA.

It has been found that stem cells could mitigate hair loss by reversing the underlying mechanisms involved in hair loss, regenerating hair follicles, and neogenesis of follicles using stem cell technologies. Having said that, the injection of cultured dermal papilla cells (DPC) in combination with cultured epithelial cells (EC) to animal models resulted in induction of hair growth [414]. Similarly, it has been shown that the addition of ADSCs to the culture of hair cells could enhance the hair follicle viability [415]. Besides, ADSC-conditioned medium was shown to double the proliferation of DPCs [415]. The application of UCSCs was also examined and found to have beneficial effects on the generation of new hair follicles and accelerating their growth in animal models [415]. Additionally, preclinical studies suggested that the administration of stem cell-derived exosomes could be advantageous for hair growth [416].

Currently, the gold standard treatment for the disease is autologous hair transplant, however, this approach also has inadequate usability because of limited material and low viability of cells achieved by the method, that should be improved [415]. In order to address this, various proposals are under investigation such as using computer-controlled stirred suspension bioreactors in order to improve the productivity and uniformity of cultured cells [415]. Furthermore, the administration of BMMCs for the treatment of refractory patchy alopecia was found to be satisfactory [417]. Also, ADSC-conditioned medium combined with a protein solution and growth factors was intradermally administered for patients with alopecia, that led to significant increase in the number of hairs [418]. Correspondingly, other studies showed the patients who had received the conditioned media of ADSCs were considerably benefited from the method, as the mean hair thicknesses and hair rates of these patients were noticeably increased [419, 420]. Another cell line which has been clinically investigated is DPC, that represented promising results, increasing the total density and cumulative diameter of hairs [421].

#### Infection

*Coronavirus disease 19*

The novel coronavirus disease of 2019 (COVID-19], which caused a global pandemic during the first half of the twenty-first century, has led many researchers from different biomedical fields to find solutions or treatments to manage the pandemic [422].

Regarding stem cell therapy, MSC administration has been presented among the therapeutic tactics used in the treatment of COVID-19, as it could present immunomodulatory effects [423-425]. Stem cell therapy and especially MSCs should be taken into consideration, either alone or in combination with other treatments, as suitable therapeutic options for the COVID-19 patients [426]. In this regard, a clinical trial showed that the administration of MSCs could significantly improve the symptoms of patients suffering from the disease, particularly, the severely affected patients [424]. In another study, allogenic ADSCs were intravenously administered for the patients under mechanical ventilation, which is found to have therapeutic effects in most cases; as the patients showed clinical, radiologic, and laboratory improvements [427]. Moreover, 3 times intravenous administration of umbilical cord MSCs for a severely ill COVID-19 patient resulted in recovery of vital signs and clinical laboratory data, as well as alleviation of pulmonary inflammation examined by computed tomography (CT) scans [425]. Similar approach, using umbilical cord MSCs, was used in a phase 1 clinical trial that led to complete recovery and discharge of the severely ill patients [428]. It is worth mentioning that exosomes derived from MSCs were also shown to recover the patients’ clinical status, restore the oxygenation capacity, and improve the cytokine storm caused by COVID-19 [429]. It seems that administration of stem cells could be a possible solution for treating the patients suffering from COVID-19, without causing serious side effects. However, clinical studies with higher populations and greater power should be conducted in the future.

#### Human immunodeficiency virus

Every year, infection with the human immunodeficiency virus (HIV) resulting in acquired immune deficiency syndrome (AIDS) affects around 2 million people worldwide and 40,000 Americans, which leads to lifelong infection, accompanied by several co-infecting diseases in addition to high mortality rates with no effective treatment up to now [430, 431]. Stem cell therapy using PSCs has the potential to continuously replace T-lymphocytes, along with the inevitable occurrence of the graft versus host effect which can subsequently eliminate the reservoir from the old-infected cells and substitute them with healthy, new cells [432-436]. Also, transplantation of stem cells has been reported effective in the management of HIV infected patients [437]. However, the true efficiency of this model is yet to be evaluated in further studies. (Table 1)

### Ethical, legal, and social considerations

There are several dilemmas and controversies surrounding stem-cell therapy and research in this regard. For example, ESC investigations are ethically and politically controversial, as it causes the destruction of human embryos. Some individuals believe that an embryo is a person with the identical rights and moral status as adults; they insist on that “human life begins at conception” because of their religious or moral beliefs [438]. From this viewpoint, captivating a blastocyst and detaching the internal cell mass to develop an ESC line is equivalent to murder [438]. Considering these contests, researchers tended to use ESC lines from frozen embryos acquired from couples who underwent infertility treatments and decided to donate their remaining frozen embryos for research purposes. Conversely to ESCs, the use of ASCs and UCSCs is less likely to face ethical challenges because their beneficial effects are already sensible in the treatment of hematological diseases. Researchers must bear in mind that informed and voluntary consent must be obtained prior to obtaining biological materials for stem cell research and clinical trials.

Other ethical issues regarding the field of stem cell therapy are the importance of reducing adverse effects, the matter of overpromising by physicians, adjustment of patient optimism and expectations levels. Moreover, one must be wary of profit making entities trying to pass these novel methods too quickly into the clinic, at the price of not understanding the basic mechanisms.

Policymakers have been discussing several issues around stem-cell therapy such as whether moral and/or religious sights should have a part in strategies, the basis, and level of the association between science and politics, and public engagement and their suitable role in the field; along with the evasion of experts to less restrictive instructions, and probability of falling behind other countries in scientific skills because of restrictive rules [439]. Religious leaders have also asserted their concerns about reproductive cloning, hybrids, and issues of ‘humanness’, and savior siblings.

Wide-ranging demands have been made globally in order to make an international regulation that has a consensus and jurisdictional consistency, regarding stem-cell therapy; hence, an international description of bioethics is needed.

## CONCLUSION

Decades of exploration showed that stem cell therapy can be a potential cure for diseases that were once thought to be untreatable. Today, patients are routinely undergoing stem cell transplants for conditions such as leukemia, lymphoma, congenital immune deficiencies, neurodegenerative, and congenital metabolic diseases. While there are numerous publications on the therapeutic uses of stem cells, both in vitro and in vivo, experimental and clinical, finding solid methods to take control of the division and differentiation processes of these cells, as well as methods of administration in different diseases has remained to be a challenge. On the other hand, in many healthcare systems, stem cell therapies are still considered as a research field rather than a therapeutic choice showing that these systems have not still put trust in this method. Hence, future efforts should be focused on removing these challenges both in the clinics and in the administration. A big step towards these goals would be large population clinical trials. If a stem cell-based treatment method can pass a clinical trial, trust will be built in it, and barriers will fall. Another suggestion is to educate society about the benefits and disadvantages of stem cell therapy. Knowledge will bring about demand and demand will motivate the healthcare system to take this therapeutic method into account.
